# Transcriptional profile of *Pseudomonas syringae *pv. phaseolicola NPS3121 in response to tissue extracts from a susceptible *Phaseolus vulgaris *L. cultivar

**DOI:** 10.1186/1471-2180-9-257

**Published:** 2009-12-14

**Authors:** Alejandro Hernández-Morales, Susana De la Torre-Zavala, Enrique Ibarra-Laclette, José Luis Hernández-Flores, Alba Estela Jofre-Garfias, Agustino Martínez-Antonio, Ariel Álvarez-Morales

**Affiliations:** 1 Departamento de Ingeniería Genética, Cinvestav-IPN Unidad Irapuato, Apdo Postal 629, CP 36821, Irapuato, Gto, México; 2 Laboratorio Nacional de Genómica para la Biodiversidad, Cinvestav-IPN Unidad Irapuato, Apdo Postal 629, CP 36821, Irapuato, Gto, México

## Abstract

**Background:**

*Pseudomonas syringae *pv. phaseolicola is a Gram-negative plant-pathogenic bacterium that causes "halo blight" disease of beans (*Phaseolus vulgaris *L.). This disease affects both foliage and pods, and is a major problem in temperate areas of the world. Although several bacterial genes have been determined as participants in pathogenesis, the overall process still remains poorly understood, mainly because the identity and function of many of the genes are largely unknown. In this work, a genomic library of *P. syringae *pv. phaseolicola NPS3121 was constructed and PCR amplification of individual fragments was carried out in order to print a DNA microarray. This microarray was used to identify genes that are differentially expressed when bean leaf extracts, pod extracts or apoplastic fluid were added to the growth medium.

**Results:**

Transcription profiles show that 224 genes were differentially expressed, the majority under the effect of bean leaf extract and apoplastic fluid. Some of the induced genes were previously known to be involved in the first stages of the bacterial-plant interaction and virulence. These include genes encoding type III secretion system proteins and genes involved in cell-wall degradation, phaseolotoxin synthesis and aerobic metabolism. On the other hand, most repressed genes were found to be involved in the uptake and metabolism of iron.

**Conclusion:**

This study furthers the understanding of the mechanisms involved, responses and the metabolic adaptation that occurs during the interaction of *P. syringae *pv. phaseolicola with a susceptible host plant.

## Background

*Pseudomonas syringae *is an important Gram-negative bacterium that infects a wide variety of plant species and causes disease symptoms ranging from leaf spots to stem cankers in agriculturally important crops. Bacteria such as *P. syringae *often live as epiphytes on the leaf surface without causing any obvious disease symptoms. However, under permissible conditions of temperature and humidity, *P. syringae *can enter the plant through natural openings such a stomata and hydathodes or via mechanical wounds [1-3]. Once bacteria enter the intercellular spaces (the apoplast), they can withstand preformed defense molecules, obtain nutrients and multiply to cause damage to the host tissue [1]. The identities of the pathogenic factors involved in these processes are largely unknown, and how they function to promote parasitism and disease is also poorly understood [4]. Adaptation of *P. syringae *to the apoplast depends on specific pathogenicity genes, such as *hrp *genes (*h*ypersensitive *r*esponse and *p*athogenicity) that encode proteins of the type III secretion system (TTSS), which in turn delivers bacterial-effectors into plant cells [5]. The induction of *hrp *genes in bacteria occurs soon after the first contact with plant tissue. Expression of *hrp *genes are detected as early as 1 h after inoculation and continue to increase for at least 6 h [6]. However, no specific plant-derivatives have been identified as inducers of *hrp *genes, and in *Ralstonia solanacearum *some evidence suggests that the full induction of *hrp *genes requires contact with plant tissues [7]. The *hrp *genes are also induced *in vitro *when bacteria are grown in minimal medium with carbon sources such as sucrose, fructose or mannitol, low pH and a low N/C ratio [6]. Minimal media with these characteristics seems to mimic some of the conditions bacteria might find within the apoplast. It has been suggested that the induction of *hrp *genes after contact with plant tissues could result from alterations in the nutritional status of the bacteria [2,6].

During the interaction with their host, it is thought that bacteria commonly detect specific plant metabolites, which are used as signals for changing their gene expression patterns, allowing them to adapt to the plant environment. Specific plant molecules such as phenolic β-glycosides, shikimic and quinic acids, and pectin oligomers have been reported to activate the expression of genes involved in toxin synthesis and cell wall degradation [8-10]. In this study, we used microarray analysis to identify genes of *P. syringae *pv. phaseolicola NPS3121 differentially expressed in response to metabolites present in plant tissue extracts [11]. Bacteria were grown on minimal medium supplemented with bean leaf extract, apoplastic fluid or bean pod extract. By using these three types of extract, we were able to identify bacterial genes that possibly facilitate the colonization of susceptible plant tissues, such as bean leaves and/or apoplastic fluid which are known targets during the infection process of *P. syringae *pv. phaseolicola NPS3121 [11,12].

## Results and Discussion

### Leaf extracts and apoplastic fluid produce highly similar transcriptional responses

**Figure 1:**
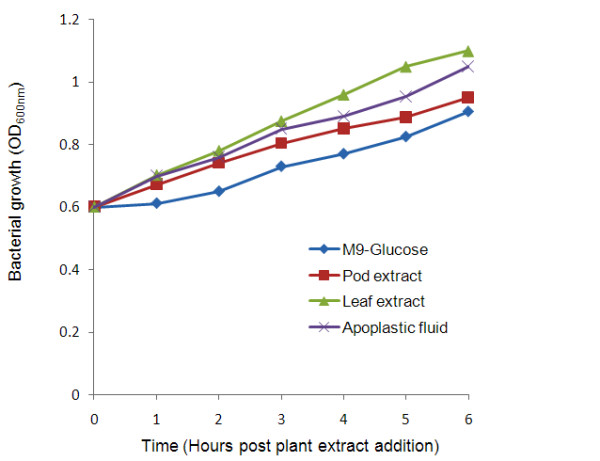
**Effects of plant extracts on cultures grown in M9 minimal media**. Growth of *P. syringae *pv. phaseolicola NPS3121 in M9 minimal medium supplemented with bean leaf extract, apoplastic fluid and bean pod extract. At mid log phase (OD_600 nm _0.6) the cultures were supplemented with 2% of plant extracts. Culture density was measured by spectrophotometry after induction during 6 hours. The bean extracts increased bacterial growth rate on supplemented media in comparison to non supplemented media.

**Figure 2:**
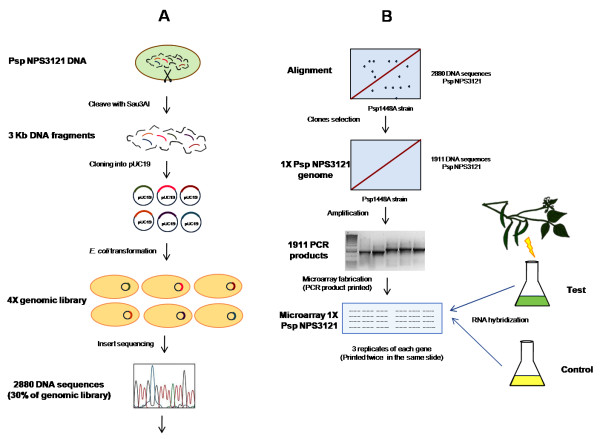
**Overview of the microarray strategy**. A library of chromosomal DNA fragments of *P. syringae *pv. phaseolicola NPS3121 (Psp NPS3121) was constructed in the pUC19 vector and introduced into the *E. coli *Top10 strain. 30% (2880 clones) of the genomic library was sequenced, aligned and annotated against the complete genome of *P. syringae *pv. phaseolicola 1448A. This strategy allowed selection of 1911 clones that provided approximately 1× coverage of the genome. The fragments of 1911 clones were amplified by PCR reaction, and the products were printed on a microarray slide. This microarray was used to identify genes that are differentially expressed when bean leaf or pod extracts and apoplastic fluid were added to the growth medium.

**Figure 3:**
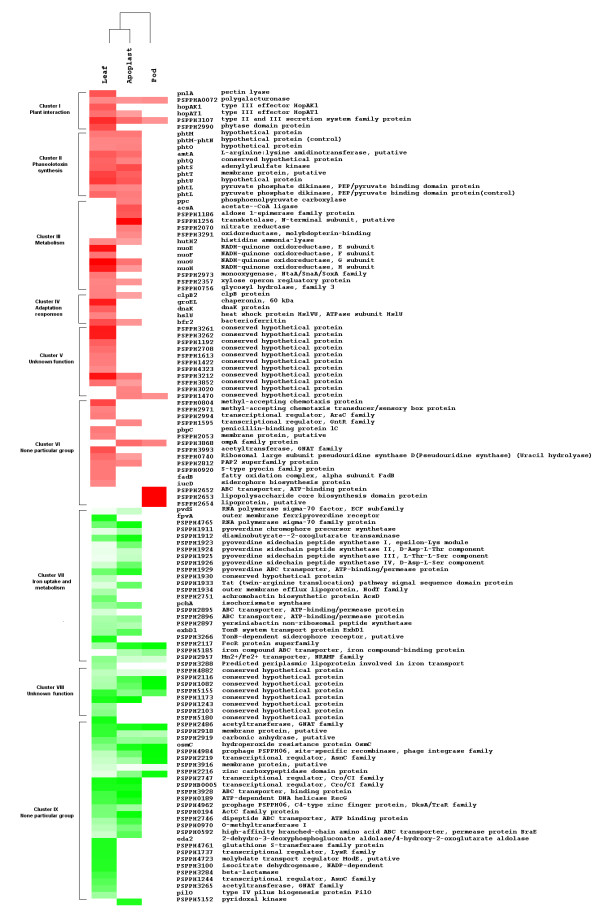
**Clustering of genes with distinct patterns of differential expression**. Differentially expressed genes with ≥ 2 or ≤ 0.5 fold change were grouped manually according to the function of their gene products, and then clustered using the complete linkage cluster algorithm. This analysis grouped genes with similar putative or known function. Red and green squares represent induced and repressed genes respectively. Intensity of color is related to magnitude of differential expression. Roman numerals represent clusters of genes mentioned in discussion of results. The complete list of the differentially expressed genes and their fold changes can be found in Additional file 1.

**Figure 4:**
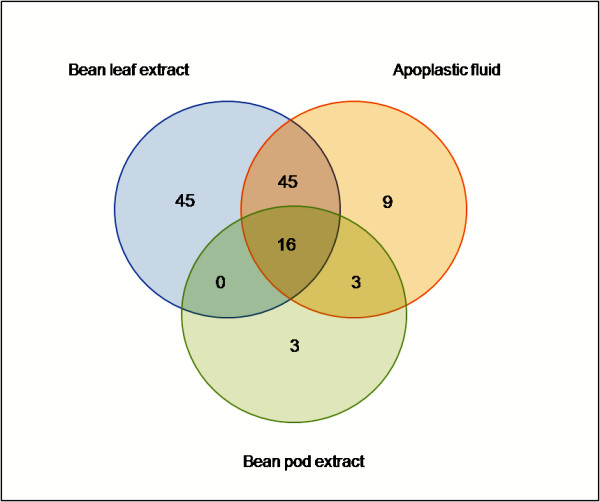
**Comparative analyses of the tested conditions**. Comparison of differentially expressed genes in *P. syringae *pv. phaseolicola NPS3121 under the effect of bean leaf or pod extract and apoplast fluid. The genes with ± 2.0 fold change were distributed as shown in Venn diagram (Tables 1 and 2). This analysis showed that bean leaf extract and apoplastic fluid had similar effects on gene transcription, 61 differentially expressed genes are being shared between both conditions.

We decided to test bean leaf and pod extracts and apoplastic fluid since these are thought to be the primary environments that *P. syringae *pv. phaseolicola encounters during infection, and in which nutrient assimilation, plant signal recognition and stress responses can occur [13,14,1,12]. To this end, *P. syringae *pv. phaseolicola NPS3121 was grown at 18°C in M9 minimal medium with glucose as a carbon source. When cultures reached the mid-log phase (OD_600 nm _0.6) bean leaf extract, apoplastic fluid or bean pod extracts were added to a final concentration of 2% and an equal amount of minimal medium was added to a control culture. The cultures were incubated for 6 hours in contact with plant extracts, the period in which expression of *hrp *genes and other plant pathogenesis related genes has been demonstrated to occur (Figure 1) [15,6,9]. RNA samples from bacteria grown in M9 minimal medium (control) and minimal medium supplemented with either bean leaf extract, apoplastic fluid or bean pod extract were labelled, mixed and used to hybridize the microarray (Figure 2 and see methods). After normalization, the genes that fall within the cut-off threshold for up-regulated genes ≥ 1.5 and for down-regulated genes of ≤ 0.6 were taken as statistically significant [16,17]. A total of 224 genes were differentially expressed in the presence of bean leaf extract, apoplastic fluid and bean pod extract. The complete list of these differentially expressed genes and their fold changes can be found in Additional file 1. However, for the rest of our discussion we focus on only 121 differentially expressed genes that fall within the traditional criteria, a cut-off threshold for up-regulated genes of ≥ 2 and for down-regulated genes of ≤ 0.5, (Table 1 and Table 2 respectively). The genes identified were grouped manually according to the function of their gene products, and then clustered based on the kind of plant extract which had produced the change in expression using the complete linkage cluster algorithm (Figure 3) [18]. Clustering shows that even though each tissue extract produced a defined transcriptional profile, apoplastic fluid and bean leaf extract had the most similar effects on gene transcription, since 50% of differentially expressed genes were common to both conditions (Figure 4), whereas for the remaining genes, the differences observed were most likely due to compositional differences between apoplastic fluid and bean leaf extract, such as sugar and nitrogen content, pH, osmolarity, phytate, and cell-wall derived molecules which could influence gene expression [19-21,14]. The bean pod extract had a less pronounced effect on the transcriptional profile with only 22 differentially expressed genes, which 16 genes are common with bean leaf extract and apoplastic fluid, corresponding to 15 and 22% of differentially expressed genes with respect to bean leaf extract and apoplastic fluid respectively (Figure 4 and see Additional file 2). The differences observed between the effects of the three types of extract suggest that each plant tissue or extract type had a defined and distinctive transcriptome expression pattern, similar to observations in previous reports for *Pectobacterium atrosepticum *grown in minimal medium supplemented with potato tuber and stem extracts [22]. Finally, due to the low response effect observed with pod extracts, it was not possible to define groups of genes dedicated to specific biological roles affected in this condition. Hence, in the following discussion we will refer exclusively to results obtained in the experiments using leaf extract and apoplastic fluid.

**Table 1:** Induced genes with ≥ 2.0 fold change in expression level FDR (p-value ≤ 0.05)

		Fold change extract/control		
		
Gene/ORF	Gene product	L	A	P
***Cluster I Plant-bacteria interaction***				

*pnlA*	pectin lyase	3.0		
PSPPH_A0072	Polygalacturonase	2.0	1.8	1.9
*hopAK1*	type III effector HopAK1	2.9		
*hopAT1*	type III effector HopAT1	2.5	1.6	
PSPPH_3107	type II and III secretion system family protein	3.7	2.6	1.8
PSPPH_2990	phytase domain protein	3.2		

***Cluster II Phaseolotoxin synthesis (Cluster Pht)***				

*phtM*	hypothetical protein	2.3	2.3	
*phtM-phtN*	hypothetical protein (control)	2.1	2.1	
*phtO*	hypothetical protein	2.1	2.1	
*amtA*	L-arginine:lysine amidinotransferase, putative	2.9	2.5	
*phtQ*	conserved hypothetical protein	2.7	2.1	
*phtS*	adenylylsulfate kinase	2.7	3.2	
*phtT*	membrane protein, putative	3.3	2.8	
*phtU*	hypothetical protein	3.5	2.9	
*phtL*	pyruvate phosphate dikinase, PEP/pyruvate binding domain protein	2.1	2.0	
*phtL*	pyruvate phosphate dikinase, PEP/pyruvate binding domain protein(control)	2.6	2.3	

***Cluster III Bacterial metabolism***				

*Ppc*	phosphoenolpyruvate carboxylase		2.2	
*acsA*	acetate-CoA ligase		3.0	
PSPPH_1186	aldose 1-epimerase family protein		2.8	
PSPPH_1256	transketolase, N-terminal subunit, putative		6.0	
PSPPH_2070	nitrate reductase		2.2	
PSPPH_3291	oxidoreductase, molybdopterin-binding		2.0	
*hutH2*	histidine ammonia-lyase	2.0	1.5	
*nuoE*	NADH-quinone oxidoreductase, E subunit	5.0		
*nuoF*	NADH-quinone oxidoreductase, F subunit	2.4		
*nuoG*	NADH-quinone oxidoreductase, G subunit	6.6	2.4	
*nuoH*	NADH-quinone oxidoreductase, H subunit	4.3	1.7	
PSPPH_2973	monooxygenase, NtaA/SnaA/SoxA family	2.3		
PSPPH_2357	xylose operon regulatory protein	2.1	1.8	
PSPPH_0756	glycosyl hydrolase, family 3	2.1		

***Cluster IV Adaptation responses***				

*clpB2*	clpB protein	2.2	1.5	
*groEL*	chaperonin, 60 kDa	4.3		
*dnaK*	dnaK protein	2.8		
*hslU*	heat shock protein HslVU, ATPase subunit HslU	2.1		
*bfr2*	Bacterioferritin	3.1	1.8	

***Cluster V Unknown function***				

PSPPH_3261	conserved hypothetical protein	4.4		
PSPPH_3262	conserved hypothetical protein	4.4		
PSPPH_1192	conserved hypothetical protein	2.8		
PSPPH_2708	conserved hypothetical protein	2.5		
PSPPH_1613	conserved hypothetical protein	2.3		
PSPPH_1422	conserved hypothetical protein	2.2		
PSPPH_4323	conserved hypothetical protein	2.0		
PSPPH_3212	conserved hypothetical protein	4.9	2.3	
PSPPH_3852	conserved hypothetical protein	2.5	1.6	
PSPPH_3020	conserved hypothetical protein		2.1	
PSPPH_1470	conserved hypothetical protein		2.2	1.9

***Cluster VI None particular group***				

PSPPH_0804	methyl-accepting chemotaxis protein	3.2		
PSPPH_2971	methyl-accepting chemotaxis transducer/sensory box protein	2.2		
PSPPH_2994	transcriptional regulator, AraC family	2.3		
PSPPH_1595	transcriptional regulator, GntR family		2.1	
*pbpC*	penicillin-binding protein 1C	2.3		
PSPPH_2053	membrane protein, putative	2.2		
PSPPH_3868	ompA family protein		2.6	2.1
PSPPH_3993	acetyltransferase, GNAT family	3.0		
PSPPH_0740	Ribosomal large subunit pseudouridine synthase D(Pseudouridine synthase) (Uracil hydrolyase)	2.6	1.6	
PSPPH_2812	PAP2 superfamily protein	2.3	2.1	
PSPPH_0920	S-type pyocin family protein	2.3		
*fadB*	fatty oxidation complex, alpha subunit FadB	2.3		
*iucD*	siderophore biosynthesis protein	2.0		
PSPPH_2652	ABC transporter, ATP-binding protein			8.7
PSPPH_2653	lipopolysaccharide core biosynthesis domain protein			10.5
PSPPH_2654	lipoprotein, putative			6.4

The table comprises all the genes that shown ≥ 2.0 fold change in expression level. **L **Bean leaf extract, **A **apoplastic fluid and **P **Bean pod extract. ORF nomenclature corresponding to 1448A reference sequenced strain. For a complete list of all statistically induced genes please consult Additional File 1.

**Table 2:** Repressed genes with ≤ 0.5 fold change in expression level FDR (p-value ≤ 0.05)

		Fold change extract/control		
		
Gene	Gene product	L	A	P
***Cluster VII Iron uptake and metabolism***				

*pvdS*	RNA polymerase sigma-70 factor, ECF subfamily	0.01	0.09	
*fpvA*	outer membrane ferripyoverdine receptor	0.47		
PSPPH_4765	RNA polymerase sigma-70 family protein	0.26	0.55	
PSPPH_1911	pyoverdine chromophore precursor synthetase	0.04	0.14	
PSPPH_1912	diaminobutyrate--2-oxoglutarate transaminase	0.26	0.53	
PSPPH_1923	pyoverdine sidechain peptide synthetase I, epsilon-Lys module	0.03	0.25	
PSPPH_1924	pyoverdine sidechain peptide synthetase II, D-Asp-L-Thr component	0.03	0.09	
PSPPH_1925	pyoverdine sidechain peptide synthetase III, L-Thr-L-Ser component	0.02	0.10	
PSPPH_1926	pyoverdine sidechain peptide synthetase IV, D-Asp-L-Ser component	0.08	0.27	
PSPPH_1929	pyoverdine ABC transporter, ATP-binding/permease protein	0.26	0.40	
PSPPH_1930	conserved hypothetical protein	0.11		
PSPPH_1933	Tat (twin-arginine translocation) pathway signal sequence domain protein	0.05	0.28	
PSPPH_1934	outer membrane efflux lipoprotein, NodT family	0.14		
PSPPH_2751	achromobactin biosynthetic protein AcsD	0.26		
*pchA*	isochorismate synthase	0.18	0.25	
PSPPH_2895	ABC transporter, ATP-binding/permease protein	0.07		
PSPPH_2896	ABC transporter, ATP-binding/permease protein	0.14	0.18	
PSPPH_2897	yersiniabactin non-ribosomal peptide synthetase	0.15	0.13	
*exbD1*	TonB system transport protein ExbD1	0.16	0.30	
PSPPH_3266	TonB-dependent siderophore receptor, putative	0.48		
PSPPH_2117	FecR protein superfamily	0.15	0.41	0.61
PSPPH_5185	iron compound ABC transporter, iron compound-binding protein		0.13	0.19
PSPPH_2957	Mn2+/Fe2+ transporter, NRAMP family	0.20	0.08	0.07
PSPPH_3288	Predicted periplasmic lipoprotein involved in iron transport	0.17		

***Cluster VIII Unknown function***				

PSPPH_4882	conserved hypothetical protein	0.11	0.06	0.05
PSPPH_2116	conserved hypothetical protein	0.12	0.32	0.65
PSPPH_1082	conserved hypothetical protein	0.14	0.28	0.63
PSPPH_5155	conserved hypothetical protein	0.37	0.20	0.31
PSPPH_1173	conserved hypothetical protein	0.46	0.66	
PSPPH_1243	conserved hypothetical protein	0.18		
PSPPH_2103	conserved hypothetical protein	0.20		
PSPPH_5180	conserved hypothetical protein	0.50		

***Cluster IX None particular cluster***				

PSPPH_2486	acetyltransferase, GNAT family	0.37	0.45	0.58
PSPPH_2918	membrane protein, putative	0.37	0.13	0.12
PSPPH_2919	carbonic anhydrase, putative	0.27	0.18	0.19
*osmC*	hydroperoxide resistance protein OsmC	0.22	0.45	0.63
PSPPH_4984	prophage PSPPH06, site-specific recombinase, phage integrase family	0.11	0.25	0.62
PSPPH_2219	transcriptional regulator, AsnC family	0.09	0.15	0.59
PSPPH_3916	membrane protein, putative	0.07	0.01	0.02
PSPPH_2216	zinc carboxypeptidase domain protein	0.04	0.20	0.54
PSPPH_2747	transcriptional regulator, Cro/CI family	0.49	0.59	
PSPPH_B0005	transcriptional regulator, Cro/CI family	0.46	0.45	
PSPPH_3928	ABC transporter, binding protein	0.34	0.63	
PSPPH_0189	ATP-dependent DNA helicase RecG	0.34	0.42	
PSPPH_4962	prophage PSPPH06, C4-type zinc finger protein, DksA/TraR family	0.24	0.16	
PSPPH_0194	ActC family protein	0.24	0.56	
PSPPH_2746	dipeptide ABC transporter, ATP binding protein	0.14	0.33	
PSPPH_0970	O-methyltransferase I	0.12	0.24	
PSPPH_0592	high-affinity branched-chain amino acid ABC transporter, permease protein BraE	0.08	0.30	
*eda2*	2-dehydro-3-deoxyphosphogluconate aldolase/4-hydroxy-2-oxoglutarate aldolase	0.43		
PSPPH_4761	glutathione S-transferase family protein	0.43		
PSPPH_1737	transcriptional regulator, LysR family	0.42		
PSPPH_4723	molybdate transport regulator ModE, putative	0.41		
PSPPH_3100	isocitrate dehydrogenase, NADP-dependent	0.40		
PSPPH_3284	beta-lactamase	0.34		
PSPPH_1244	transcriptional regulator, AsnC family	0.30		
PSPPH_3265	acetyltransferase, GNAT family	0.27		
*pilo*	type IV pilus biogenesis protein PilO	0.16		
PSPPH_5152	pyridoxal kinase		0.43	

The table includes genes that shown ≤ 0.5 fold change in expression level. **L **Bean leaf extract, **A **apoplastic fluid and **P **Bean pod extract. ORF nomenclature corresponding to 1448A reference sequenced strain. For a complete list of all statistically repressed genes please consult Additional File 1.

### Bean leaf extract and apoplastic fluid induce bacterial genes involved in the first stages of plant infection

**Figure 5:**
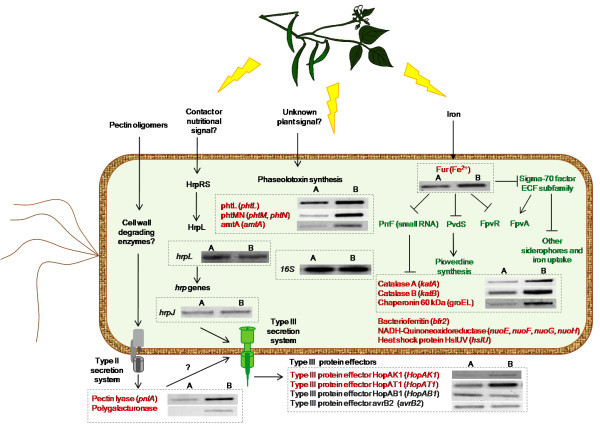
**Functional analysis of the results of microarray profiles**. Red and green letters represent induced and repressed genes respectively. Gray words represent genes constitutively expressed under our study conditions (name of genes or their identifiers are in parenthesis). We propose that induction of some genes is related to the presence of host components in the medium (leaf and apoplast). Similarly, repression of genes involved in iron acquisition, suggests that host extracts are a non-limiting source of this element. The figure also shows results of RT-PCR validation; control culture without extract (A, at the left) and test culture with bean leaf extract (B, to the right). Nine up-regulated genes were selected for RT-PCR analysis. The independent determination of transcript levels using RT-PCR analysis was congruent with the microarray data. Additionally we included genes involved in protection against oxidative stress such as catalase A (*katA*), and genes involved in TTSS (*hrpJ, HopAB1, avrB2*), which in the case of the latter are also included as controls in the microarrays and the *fur *gene.

Phytopathogenic bacteria possess a large number of genes that allow them to multiply and cause disease on plants. Many of these genes are induced only *in planta *or in the presence of host components, suggesting that gene expression is regulated by signals that bacteria receive from the plant tissue. In this study, we identified a cluster of six genes that includes genes already known to be induced during the interaction of the bacteria with its host plant and which could be used as positive controls in this study (Figure 3 and see below). Four genes of this group; pectin lyase, polygalacturonase and the type III effector proteins HopAK1 and HopAT1 were previously classified as virulence factors in the annotated genome of *P. syringae *pv. phaseolicola 1448A [23]. As shown in Figure 5 the expression levels of the type III effector proteins HopAK1 and HopAT1 increase significantly under the effect of bean leaf extract, suggesting the presence of an inducing signal in this extract. It seems that M9 minimal medium mimic some of the conditions to what the pathogen encounters in the apoplast, moreover it was recently shown by Rico and Preston that apoplast extracts support higher growth while promoting TTSS expression than synthetic minimal media [6,14]. This supports the idea that apoplast extracts provide more nutrients than minimal media with glucose as carbon source (Figure 1). [14]. As we are using this medium, we did not expect to find changes in the levels of expression of genes needed for TTSS assembly, for example *hrpJ *gene as shown in figure 5. However, changes were observed in the effector proteins HopAK1 and HopAT1 that could be attributed to the presence of specific signal molecules in both the leaf extract and the apoplast fluid. It has been demonstrated that type III effector proteins are translocated through the TTSS directly into the cytosol of the host cell, where they interfere with or modulate host cell processes to facilitate bacterial multiplication, invasion and disease [24-26]. Genes encoding pectin lyase and polygalacturonase were also up-regulated (Figure 5). Previous studies demonstrated that pectin lyase and polygalacturonase are both induced in plant tissues or *in vitro *cultures that contain plant extracts [27,28,4,22]. Both, pectin lyase and polygalacturonase are involved in pectin degradation, and possibly facilitate the assembly of functional type III secretion complexes [29-31]. In *P. syringae *strains, pectin lyase, polygalacturonase and type III effector proteins with a pectate lyase domain, such as HopAK1, are found in some pathovars, however little is known about their role and contribution to pathogenicity [32-35]. The four genes discussed above show a *hrp box *motif in their regulatory region; this element is recognized or bound by HrpL, an alternative RNA polymerase sigma factor that regulates the expression of many genes involved in pathogenesis and virulence [36,4]. Thus, if this group of genes is transcribed by a common sigma factor, it makes sense that it is found to be up-regulated under these conditions. However RT-PCR analysis showed that *hrpL *is also expressed in M9 without plant extracts therefore some possibilities are that an additional regulator is necessary to activate these genes or some anti-sigma could be inactivated in this precise condition. Definitively more studies are necessary to find the mechanism of transcription of this group of genes by HrpL (Figure 5). In addition, cluster I contains a gene that encodes a protein with a secretin N-domain that is closely related to bacterial type II and III secretion system proteins, which export proteins from within the bacterial cell to the extracellular matrix and/or into target host cells [25]. Leaf extract also induces a gene encoding a protein with a phytase domain, most likely involved in the hydrolysis of the phytate present in the bean leaf extract [37-39].

Bean leaf extract was obtained by maceration, where bean leaves were pulverized and homogenized in water. During this process it is probable that plant compounds such a phytate and cell wall derived pectin oligomers are solubilized within the extract. If these compounds are present in the extract, it makes sense that genes involved in phytate and pectin degradation are up-regulated on exposure to bean leaf extract, contrary to the effect observed with apoplast extract. Apoplastic fluid was isolated by infiltration-centrifugation procedures, a method widely used to obtain apoplastic fluid with minimal cytoplasmic contamination, which ensures that cell-wall fragments, plant debris, or any others factors are excluded [40,9,14,20,21]. Thus, apoplastic fluid does not contain cell wall derivatives, phytate or a signal(s) capable of inducing genes involved in phytate and pectin degradation correlating well with the results obtained (Table 1, Figure 3).

### Bean leaf extract induces the expression of genes involved in the synthesis of phaseolotoxin

Cluster II contains genes involved in phaseolotoxin synthesis, the production of which is temperature dependent, with an optimum at 18°C (Figure 3). The phaseolotoxin cluster (*pht *cluster) is composed of 23 genes organized in five transcriptional units, two monocistronic and three polycistronic [41]. Since our study was performed at 18°C, the optimal temperature for toxin production, it was expected that the genes of the *pht *cluster would be expressed in control and test cultures. However, seven genes of the *phtM *operon, *phtM, phtO, amtA, phtQ, phtS, phtT, phtU; *and *phtL *showed increased levels of transcription in the presence of bean leaf extract and apoplastic fluid compared to M9 medium alone (Table 1). Nevertheless, this was not the case for bean pod extract. This result indicates that in addition to the requirement of low temperature, for the optimum expression of phaseolotoxin, specific plant components present in leaf and apoplast are probably also required. Analysis of reverse transcription of *phtL*, intergenic region of *phtMN*, and *amtA*, confirmed that expression of these genes is enhanced by components present in leaf extract (Figure 5). Additionally, two genes, *phtB *and *desI*, which belong to the *phtA *and *phtD *operons respectively, showed a 1.5 fold increase in expression, values that are statistically significant on the basis of the microarray analysis (see Additional file 1 for *phtB *and *desI *genes).

Previous studies demonstrated that secreted plant signals such as phenolic β-glycosides induce genes involved in syringomycin synthesis in *P. syringae *pv. syringae [42,43,8]. Likewise, in *P. syringae *pv. tomato DC3000, the coronatine biosynthetic genes were strongly induced by crude extracts and apoplastic fluid from tomato leaf. The active compounds responsible for this induction were identified as shikimic, quinic, malic and citric acids, but it is unclear how specifically these environmental signals influence the transcription of coronatine biosynthetic genes [9]. In *P. syringae *pv. phaseolicola, no plant signal that induces phaseolotoxin synthesis has been identified so far. Our results suggest that some of these signals might be present in bean leaf extract and apoplastic fluid. In contrast, no changes were observed in the expression pattern of these genes when bacteria were exposed to bean pod extract with the exception of the *argK *gene whose expression decreased (see Additional file 1). The *argK *gene encodes an ornithin-carbamoyl-transferase (OCTase) involved in bacterial immunity against its own toxin and is expressed at 18°C in coordination with phaseolotoxin synthesis [44]. The reason why expression of this gene decreased in the presence of pod extract is unclear at this moment; however, it has been shown that expression of this gene is only partially dependent on temperature, as a small signal molecule resembling carbamoyl phosphate as inducer is also required [45]. On the other hand, bean pods infected with *P. syringae *pv. phaseolicola do not show the characteristic chlorotic halo caused by the action of phaseolotoxin [12]. It is unclear whether this phenomenon might be due to an unknown bean pod signal that inhibits phaseolotoxin synthesis.

### *P. syringae *pv. phaseolicola NPS3121 adapts its metabolism to take advantage of nutrients provided by its host plant

*P. syringae *pv. phaseolicola NPS3121 was grown in M9 minimal medium supplemented with either bean leaf extract, apoplastic fluid or bean pod extract. The growth of the cultures was monitored by optical density measurements during the induction period until the beginning of the late-log phase. The bean extracts increased bacterial growth rate on supplemented media in comparison to non-supplemented media, suggesting that plant extracts contained nutrients that enhanced the growth of the bacteria (Figure 1). Apoplastic fluid induces genes involved in carbon and nitrogen metabolism suggesting that the bacteria may use carbon and nitrogen sources present in apoplast fluid. In cluster III we classified genes involved in bacterial metabolism. Four genes *ppC, acsA*, PSPPH_1186, PSPPH_1256 involved in either, carbon fixation, glycolysis, pyruvate metabolism and/or the pentose phosphate pathway were induced, and are probably related to assimilation of sucrose, mannose, glucose or fructose, which are the most common sugars in the plant apoplast (Figure 3) [46,21]. With respect to nitrogen metabolism, our results showed that apoplastic fluid induces two genes one encoding a nitrate reductase and the other an oxidoreductase-molybdopterin-binding protein. These proteins are involved in converting nitrate to nitrite, which can be further reduced to ammonia (Figure 3 and see Additional file 1 for oxidoreductase-molybdoptering-binding protein). The induced gene *hutH2 *encodes a histidine ammonia-lyase, which catalyzes the first step in the degradation of histidine to produces urocanic acid. Both ammonia and urocanic acid are incorporated in glutamate metabolism, suggesting that this pathway is active when bacteria were exposed to apoplastic fluid. In addition, the gene *gabP *encoding a permease for γ-aminobutyric acid (GABA) was induced with apoplastic fluid (see Additional file 1). GABA is the most abundant amino acid in the plant apoplast and is used as a nitrogen source by *P. syringae *pv. phaseolicola 1448A and other related pathovars [14,20,46].

On the other hand, the genes involved in carbon and nitrogen metabolism are not highly expressed under the effect of bean leaf extract. We speculate that the leaf extract is capable of providing most of the carbon and nitrogen metabolic intermediates required to sustain bacterial growth, without the need to express genes involved in the synthesis of such compounds.

Despite the fact that bean pod extract has a positive effect on bacterial growth; a minimal effect on genes involved in metabolism was obtained in comparison with the other extracts. It is possible that differences in nutrient content, pH, catabolite repression, or tissue specificity promote differential expression between whole leaf tissue (including apoplast) and pod tissue [47].

Cluster III also includes the *nuoE, nuoF, nuoG *and *nuoH *genes, all of which are members of the *nuo *operon. This operon encodes the first enzyme of the respiratory chain, NADH-dehydrogenase [48,49,23]. The *nuo *operon of *P. syringae *pv. phaseolicola 1448A contains 13 genes, however in our microarray only the four genes mentioned above are present. The induction of these four genes suggests that all the other genes of the *nuo *operon were induced to maintain levels of metabolic activity in the bacteria according to energy demand.

### Bean leaf extract and apoplastic fluid induce genes related to adaptation responses

Cluster IV includes a group of four genes, three of which: *clpB2, groEL*, and *dnaK *encode chaperones, and *hsIU *which encodes a heat shock protein (Figure 3). Chaperones are involved in numerous bacterial processes such as, folding newly synthesized proteins, protein secretion, prevention of aggregation of proteins on heat shock, and reparation of proteins that have been damaged or misfolded by stresses. Induction of genes encoding chaperones is perhaps an indication of high protein re-flux as a product of an active or adaptive metabolism [50]. Another possibility is that these chaperones are required for assembling the secretion systems mentioned above which export products induced in response to bean leaf extract [51].

Analysis of reverse transcription showed that leaf extract induced two genes involved in protection from oxidative stress, *katA *and *katB *which encode catalases A and B respectively and are associated with the detoxification of reactive oxygen species produced as a consequence of aerobic metabolism, or the presence of iron and/or toxic molecules in the plant extracts (Figure 5) [52,53]. Most bacterial catalases require haem groups for catalytic activity; the final step of haem synthesis is catalyzed by ferrochelatase, which condenses Fe2+ into protoporphyrin IX. In P. aeruginosa, the cellular source of iron required for haem assembly is the protein bacterioferritin A, encoded by the *bfrA *gene, that is required as an iron supplier for the haem group of *KatA *and thus for protection against H_2_O_2 _[54]. Our results show that gene *bfr2 *encoding an iron storage bacterioferritin was induced under the effect of bean leaf extract and apoplastic fluid, which may supply iron for catalase activity (Figure 3, Table 1).

In summary, the increased growth in media supplemented with plant extracts can be associated with nutrient assimilation and active metabolism. In these conditions we identified genes involved in carbon and nitrogen utilization, chaperones, heat shock proteins and those involved in protection against oxidative stress (Table 1). Some of the identified genes such as heat shock proteins, bacterioferritin, and genes involved in defense against oxidative stress are positively regulated by the Ferric uptake regulator protein (Fur) [55]. These findings suggest that aerobic metabolism is active during contact with plant tissues, as will be discussed below in the section describing repressed genes (Figure 5).

Additionally 16 genes were grouped into two clusters. Cluster V includes 11 poorly characterized genes; seven of these are preferentially induced by leaf extract and may have functions related to responses to signal molecules present in the extracts. Some other induced genes that could not be classified as being involved in a particular biological process, were included in Cluster VI, two genes involved in chemotaxis, two transcriptional regulators of the AraC and GntR families and four genes which may be related to membrane biogenesis (Figure 3 and Table 1).

### Bean leaf extract and apoplastic fluid down-regulate genes involved in iron uptake and metabolism

Cluster VII was the largest cluster and contained 24 genes repressed in response to bean leaf extract and apoplastic fluid (Figure 3). Thirteen of these genes are known or hypothesized to be associated with pyoverdine production. This group includes *pvdS*, an extracytoplasmic sigma factor (ECF) needed for the transcription of genes for pyoverdine synthesis, a ferripyoverdine receptor (FpvA) involved in binding of iron-siderophore complexes in *P. aeruginosa *and a gene encoding a sigma-70 family protein with 34% identity (aa) to sigma factor FpvI, which is needed for *fpvA *transcription [56,57]. In this cluster there are also five genes associated with biosynthesis of achromobactin and yersiniabactin, the secondary siderophores in *P. syringae *pv. syringae B728a and *P. syringae *pv. tomato DC3000 respectively (Table 2) [58,59]. Two of these genes whose products belong to an ABC transporter system are located close to genes for yersiniabactin synthesis on the chromosome and are probably involved in transporting this siderophore [23]. Two genes of the TonB transport system required for active transport of iron-siderophore complexes, and another gene encoding the regulatory protein (FecR) and proteins involved in iron uptake/transport are also included in this group (Table 2) [60]. Many genes in this cluster have been shown to be regulated by Fur in *P. aeruginosa*. In this bacterium Fur has been revealed as a master regulator of iron homeostasis. Fur acts as a general repressor of iron uptake genes when the amount of their iron co-repressor (Fe^2+^) reaches a threshold level (Fur-Fe^2+^). In contrast, under iron-limiting conditions, Fur repression is relieved and transcription can occur. In *P. aeruginosa *Fur represses the transcription of the *pvdS *and *fpvI *genes, both encoding extracytoplasmic sigma factors (ECFó). PvdS and FpvI are needed for transcription of all pyoverdine related genes and the pyoverdine receptor (FpvA) respectively (Figure 5) [61,55]. The PvdS sigmulon is conserved among the fluorescent pseudomonads, including plant pathogens of the *P. syringae *group [57]. In *P. syringae *pv. phaseolicola 1448A, the cluster associated with pyoverdine synthesis contains 29 genes, of which 13 genes were printed in our microarray, including orthologs of *fpvA *and *pvdS *[23,57]. All of these genes were repressed under the tested conditions (Table 2). Although the gene encoding the Fur repressor was not printed in our microarray, its functional status can be inferred as active on the basis that genes regulated by this protein are repressed. Moreover analysis of reverse transcription of the *fur *gene confirmed that it is up-regulated under our conditions (Figure 5). These results suggest that plant extracts contain the co-repressor (Fe^2+^) at non-limiting concentrations and this causes a strong repression of iron responsive genes possibly through a regulatory cascade similar to that found in Fur-mediated repression in *P. aeruginosa *(Figure 5) [55].

It is also known that under conditions of iron-sufficiency the Fur protein represses two small RNAs in *P. aeruginosa *(PrrF1 and PrrF2), which in turn control negatively, at post-transcriptional level, the expression of genes for the pathways that are associated with the availability of large amounts of iron [62]. Thus, the positive regulation of Fur is mediated through its negative regulation of the negative regulatory RNAs (repressing the repressors). PrrF-regulated genes are derepressed under iron-sufficient conditions and are involved in a wide range of metabolic activities such as iron storage (*bfr2*), defense against oxidative stress (*katA, katB*), induction of heat shock proteins (*hsIU*), carbon metabolism and electron transport (*nuoE, nuoF, nuoG, nuoH*) in aerobic conditions [55]. All these observations are congruent with the metabolic status of the bacteria, produced in our study conditions, as mentioned above in the induced genes section (Figure 3, Figure 5). Two putative homologous *prrF *sequences were found in *P. putida*, *P. fluorescens*, and *P. syringae*, suggesting that the small RNAs (PrrF1 and PrrF2) are conserved among the pseudomonads [62]. A search in the *P. syringae *pv. phaseolicola 1448A genome revealed an intergenic region with approximately the same length and 84% and 83% nucleotide identity with PrrF1 and PrrF2 respectively. In our study many genes regulated by PrrF in other pseudomonads were also up-regulated, suggesting that this positive effect might also be mediated by the Fur protein and the PrrF sRNA which regulate genes involved in carbon metabolism, bacterioferritin, catalase production and electron transport (Figure 5) [55,62].

## Conclusions

The apoplast is the first point of contact of *P. syringae *pv. phaseolicola during the infection of the plant. However, apoplastic fluid will not completely mimic the conditions present *in planta*, which include the interaction with intact plant cell walls and plant metabolites that are only produced as a reaction to the presence of the bacteria. Here we investigate the physiological adaptation of *P. syringae *pv. phaseolicola NPS3121 when grown in the presence of leaf and pod extracts and apoplastic fluid. The greatest number of genes showing significant changes in expression levels was obtained under the effect of bean leaf extract and apoplastic fluid, in contrast with bean pod extract, which affected only a few genes. These results demonstrate that each tissue or extract type produces a defining and distinctive transcriptional pattern in the bacteria and that the shared expression profiles were correlated with the biological relationship of the extract type (leaf and apoplastic fluid).

Up-regulated genes include those encoding cell wall degrading enzymes, secretion system proteins (TTSS), proteins involved in phaseolotoxin synthesis, carbon and nitrogen metabolism, aerobic respiration (*nuo *operon), adaptation responses and protection against oxidative stress. On the other hand, some down-regulated genes are clearly involved in iron uptake and transport, suggesting that host extracts provide enough iron for bacterial growth.

We speculate that under the experimental conditions of this study bacteria produce reactive oxygen species as a consequence of aerobic metabolism. High iron concentration (of the plant extract) during aerobic respiration can lead to interactions that generate the highly reactive oxygen species that can damage a variety of cellular components. Therefore, iron metabolism must be carefully balanced in terms of acquisition and storage. The results showed that bacteria repress genes involved in iron acquisition, induce iron dependant enzymes and iron storage proteins (bacterioferritin) that provide the cofactor Fe^2+ ^for catalase, which is involved in protection against oxidative stress. These responses allow *P. syringae *pv. phaseolicola NPS3121 to adapt to media supplemented with plant extracts.

In addition, the results demonstrate that for many genes, a significant increase in expression is probably due to plant signal molecule(s) found in bean extracts. The role of some of these gene products such a pectin lyase, polygalacturonase and TTSS proteins during the first stages of the plant-bacterial interaction and the role of phaseolotoxin in virulence has previously been reported. Furthermore, this study suggests that to obtain information of genes required for the late stages in the infective process, other approaches such as gene expression analysis in infected tissue may be required. This type of analysis could provide information about processes occurring during metabolic adaptation to host tissue, disease development ranging from first stages to the development of symptoms and bacterial physiology influenced by responsive factors such as antimicrobials and other defensive metabolites inside the plant cell.

## Methods

### Assembly of a DNA microarray of *P. syringae *pv. phaseolicola NPS3121 (see Figure 2)

Genomic DNA from *P. syringae *pv. phaseolicola NPS3121 was isolated as described previously [63], partially digested with Sau3AI and run on a continuous sucrose gradient to recover fragments with an average size of 3 kbp. The genomic fragments were ligated into the plasmid vector pUC19 (Invitrogen, California, USA) previously digested with BamHI, and the ligation mixture was used to transform *Escherichia coli *TOP10 cells (Invitrogen, California, USA). Transformants were transferred to 96-well microplates, grown overnight and plasmids were recovered. A total of 9792 recombinant clones were obtained with an average insert size of 2.6 kbp giving an estimated 4× coverage of the *P. syringae *pv. phaseolicola NPS3121 genome whose size is reported to be 5640 Mpb [64]. Around 30% of the genomic clones were randomly selected and partially sequenced in a single direction using the forward M13-primer (5'-CCCAGTCACGACGTTGTAAAACGAC) by the Sanger method. 2880 sequences with an average size of 531 pb were obtained. Using the MUMmer system each sequence was aligned and annotated against the complete genome sequence of *P. syringae *pv. phaseolicola 1448A [23]. This strategy allowed us to select those clones that provided approximately 1× coverage of the genome, eliminating redundancy and providing information regarding the identity of the 5' end of each clone. From these, 1911 clones were amplified by PCR in a 100 μl PCR reaction containing 10 μM M13 forward and reverse primers (as above and 5'-AGCGGATAACAATTTCACACAGGAA), 10 μl of 10× PCR buffer, 50 mM MgCl_2_, 10 mM dNTP mix and 1 unit of *Taq *DNA polymerase. Thirty cycles of: denaturation at 94°C for 30 s, annealing at 60°C for 30 s, and extension at 68°C for 3 min were performed, followed by 5 min of final extension at 68°C. Amplified products were visualized on ethidium bromide-stained agarose gels. These PCR products were purified, dissolved in water, and quantified using a ND-1000 Spectrophotometer (NanoDrop Technologies, Wilmington, DE, USA). The DNA concentration for each sample (average size 2.4 kbp) was adjusted to 240 ng/μl in 1× spotting solutions (Micro Spotting Plus, ArrayltTM, Sunnyvale, CA), and then spotted onto Gamma Amino Propyl Silane coated slides (Corning Inc., NY, U.S.A.) using the Virtek Chiprender Professional Arrayer at 20°C and 60% humidity. As controls, PCR products for genes involved in the synthesis of the type III secretion system (*hrpRS, hrpTU, hrpOP, hrpJ, virPphA, avrPphC, avrPphD, avrPphE*), phaseolotoxin synthesis (*argK, phtA, phtD, desI, phtL, phtMN, amtA*), quorum sensing (*ahlI, ahlR, algD*), global regulators (*rpoD, gacA, rpoN, gacS, rsmA*), and lucidea universal ScoreCard controls (GE) were printed on the microarray to validate, filter and normalize data. All samples were printed in triplicate in a contiguous arrangement of 12 grids of 24 rows × 24 columns. The microarray was printed twice on the same slide for a total 6 replicates for each fragment. To further check the quality of the printed microarrays, a quality control assay was performed. To this end, *P. syringae *pv. phaseolicola NPS3121 was grown at 18°C in minimal M9 medium until it reach the late-log phase (OD_600 nm _0.95-1.0), RNA was isolated, and cDNA was synthesized and labelled with either dUTP-Cy5 or dUTP-Cy3. The cDNAs were used as probes to hybridize the microarray. The Cy3 and Cy5 signals were quantified, and the corresponding analyses were performed as described below in the microarray analysissection. Most spots printed on the DNA microarray showed uniform intensities of fluorescence when hybridized with RNA of strain NPS3121 grown in a single condition. Accordingly, when the means of signal intensity of the Cy5 probe were plotted against those of the Cy3 probe, a curve with slope 1 was obtained. Most signals were found near the diagonal, indicating that most of the genes were constitutively expressed (data not shown). After the quality control had shown that the DNA microarray results were reliable, we aimed to characterize the changes in the transcriptional profile of *P. syringae *pv. phaseolicola NPS3121 under the effects of bean leaf extract, apoplastic fluid, and bean pod extract.

### Preparation of bean leaf and pod extracts, and apoplastic fluid

Bean plants (*Phaseolus vulgaris *L. cv. Canadian Wonder) were grown in a controlled environmental chamber for 3 to 4 weeks (16 h light/8 h dark [25°C]). Leaf and pod extracts were obtained according to the methodology described by Li and collaborators [9], using 1 g of tissue mixed with 2 ml of water. Apoplastic fluid was obtained from 3 to 4 week-old bean plants by a method described previously [40,9].

### Bacterial growth conditions and RNA extraction

*P. syringae *pv. phaseolicola NPS3121 was inoculated in 20 ml of M9 minimal media with glucose (0.8%) as carbon source and cultured overnight at 28°C. The cells were washed with minimal medium and inoculated into 200 ml of M9 minimal medium at OD_600 nm _0.1. The bacteria were grown at 18°C until the mid-log phase (OD 600_nm _0.6). The culture was then split into two equal parts. One of which was induced with 2% of bean leaf or pod extract or apoplastic fluid and to the other an equal amount of minimal medium was added as control. Each culture was incubated for 6 h at 18°C, until the beginning of late-log phase and the cells were then recovered by centrifugation. Total RNA was isolated from these cultures using Trizol reagent as recommended by the manufacturer (Invitrogen, California, USA). A second step of purification was performed using RNeasy MinElute spin columns (Qiagen, Valencia, CA) to remove any contaminating DNA. RNAs were eluted in 50 μl of diethylpyrocarbonate (DEPC)-treated water and their concentration was determined using the NanoDrop spectrophotometer. RNA integrity was checked by analytical agarose gel electrophoresis.

### Synthesis of fluorescently labelled cDNA from *P. syringae *pv. phaseolicola NPS3121 total RNA

First-strand cDNA was synthesized using the CyScribe First-Strand cDNA Labelling kit (Amersham Biosciences). Thirty μg of total RNA was mixed with 3 μl of random nonamers, 0.5 μl anchored oligo (dT), 1 μl score card Spike mix control or test, and 1 μl score card utility mix (in a final volume of 11 μl). The RNA sample was heated at 70°C for 5 min. Reactions were held at room temperature for 10 min to allow the primers and the RNA template to anneal. To each reaction, the following were added: 4 μl of 5× first strand buffer, 1 μl of 1 mM Cy5-dUTP or Cy3-dUTP, 2 μl of dithiothreitol 100 mM, 1 μl of dUTP nucleotide mix and 100 U of Superscript II reverse transcriptase. cDNA synthesis was performed at 42°C for 2 h in the dark and then the RNA template was hydrolyzed by incubation with 2 μl of 2.5 N NaOH at 37°C for 15 min. The reaction was neutralized by adding 10 μl of 2 M HEPES. The labelled cDNA was purified using the CyScribe GFX purification Kit as recommended by the manufacturer (Amersham Biosciences). The incorporation of Cy3 or Cy5 nucleotides into first-strand cDNA was quantified with the NanoDrop equipment and samples were finally stored at -20°C before use.

### Microarray hybridizations

Printed microarray slides were hydrated with distilled water steam and fixed with a UV cross linker at 1200 J, then denatured in boiling water for 2 min, immersed in 95% ethanol and dried. The slides were prehybridized at 45°C for 1 h in 5× SSC, 0.1% SDS, 1% BSA. They were then washed twice for 5 min in 0.1× SSC and 30 s in 0.01× SSC, dried and used directly for hybridization. The labelled probes (10 μl) were added to 10 μl hybridization buffer (containing 3× SSC, 0.1% SDS, 1% BSA) and 10 μl of formamide. Probes were denatured at 95°C for 5 min and applied onto the genomic array slide, covered with a cover slip (Hybri-slips, Sigma-Aldrich Co. St Louis U.S.A.) and hybridized at 45°C for 16 h. After hybridization the slides were washed sequentially for 5 min each in 2× SSC-0.1% SDS, 0.1× SSC-0.1% SDS, 0.1× SSC, and 0.01× SSC. The slides were dried and fluorescent signals were scanned using an Axon Genepix 4000B scanner at a resolution of 10 μm adjusting the laser and gain parameters to obtain similar levels of fluorescence intensity in both channels. Each microarray experiment was repeated six times (two technical replicates with the same RNA samples and three biological replicates using RNA isolated from a different culture).

### Analysis of DNA microarray data

Spot intensities were quantified using Axon GenePix Pro 6.0 image analysis software. First, an automatic spot finding and quantification option of the software was used. Subsequently, all spots were inspected individually and in some cases, the spot diameters were corrected or the spots were removed from the analysis. The mean of the signals and the median of backgrounds were used for further analysis. Raw data were imported into the R 2.2.1 software [65]. Background signals were subtracted using the Robust Multichip Analysis "RMA" [66] whereas normalization of the signal intensities within slides was carried out using the "printtiploess" method and the LIMMA package [67,68]. Normalized data were log2 transformed and then fitted into mixed model ANOVAs using the Mixed procedure [17,18]. The *p*-values of the bean extract effects were adjusted for by the False Discovery Rate method "FDR" [69]. Changes in signal intensity of ± 1.5-fold or higher/lower between treatments and controls were highly significant (FDR, p-value ≤ 0.05), however we focus only in differential expressed genes that fall in the more traditional criteria, which is the cut-off threshold for up-regulated (≥ 2) and down-regulated genes (≤ 0.5). The genes were subject to cluster analysis with Gene Cluster 3.0, using the uncentered Pearson correlation and complete linkage clustering. Results were visualized with Treeview as described by Eisen and collaborators [18].

### Microarray validation by Reverse transcription-PCR analysis

RT-PCR analysis was carried out to validate the array hybridization data. RT-PCR analysis was performed for nine up-regulated genes under the effect of bean leaf extract. These RT-PCR experiments involved independent biological experiments from those used for microarray analysis. DNA-free RNA was obtained and checked for integrity in an agarose gel, 200 ng of total RNA were used for reverse transcription (RT) and PCR using the SuperScript one-step kit (Invitrogen, California, USA). A list of the primers used in this analysis is available on request. Controls used for each set of primers were (i) PCR without the reverse transcription step to verify the absence of DNA, (ii) RT-PCRs performed without RNA templates to detect any contaminating DNA/RNA, (iii) PCRs performed using chromosomal DNA as a template to ensure primer fidelity, and (iv) amplification of a portion of the 16S rRNA operon using suitable primers as an internal control of the reaction. The RT reaction was performed at 50°C for 30 min, followed by PCR amplification at 94°C for 2 min for 1 cycle; 94°C for 35 s, 55-58°C for 30 s, and 72°C for1.0 min for 28 cycles; and 72°C for 10 min for 1 cycle.

### Microarray data accession

The microarray data from this study is available on the GEO database at http://www.ncbi.nlm.nih.gov/geo under series GSE14625, GSE14983, and GSE14998.

## Authors' contributions

AH-M contributed to experimental design; microarray fabrication, performed experiments, analyzed the data and drafted the manuscript. ST-Z participated in the design of the study and microarray fabrication. EI-L contributed to experimental design, microarray fabrication, analyzed microarray data and performed statistical analysis. JLH-F participated in the design of the study. AEJ-G participated in the design of the study. AM-A contributed to interpretation of data and revision of the manuscript. AA-M conceived the study, contributed to experimental design and edited the manuscript.

## Supplementary Material

Additional file 1 **Table of differential expressed genes**. This Excel file contains all differentially expressed genes under effect of bean leaf, pod extract, and apoplastic fluid. The table contains 224 genes that showed ± 1.5 fold change in expression level. Comparative analysis was performed and the genes were grouped in accordance with similar responses. The group A comprises differential expressed genes in response to three plant extracts. Group B include genes in response to bean leaf extract and apoplastic fluid. Group C include genes in response to apoplastic fluid and bean pod extract. The group D, E and F comprises genes in particular responses to bean leaf extract, apoplastic fluid and bean pod extract respectively. The file includes a Venn diagram that shows the relations between the responses to three plant extracts. Click here for file

Additional file 2 **Table of differential expressed genes with the more stringent cut-off**. The table contains 121 genes with ± 2.0 fold change in expression level. These genes were grouped according to the function, and then clustered based on the kind of plant extract using the complete linkage cluster algorithm. The cluster of induced and repressed genes that are discussed in manuscript and a comparative Venn diagram are also shown. Click here for file

## References

[B1] Hirano SS Upper CD Bacteria in the leaf ecosystem with emphasis on *Pseudomonas syringae *: A pathogen, ice nucleus, and epiphyte Microbiol Mol Biol Rev 2000 64 624 653 10.1128/MMBR.64.3.624-653.2000 10974129 PMC99007

[B2] Jin Q Thilmony R Zwiesler-Vollick J Sheng-Yang H Type III protein secretion in *Pseudomonas syringae* Microb Infect 2003 5 301 310 10.1016/S1286-4579(03)00032-7 12706443

[B3] Bretz JR Hutcheson SW Role of type III effector secretion during bacterial pathogenesis in another kingdom Infect Immun 2004 72 3697 3705 10.1128/IAI.72.7.3697-3705.2004 15213109 PMC427461

[B4] Boch J Joardar V Gao L Tara LR Lim M Kunkel BN Identification of *Pseudomonas syringae *pv. tomato genes induced during infection of *Arabidopsis thaliana* Mol Microbiol 2002 44 73 88 10.1046/j.1365-2958.2002.02877.x 11967070

[B5] Alfano JR Collmer A Bacterial pathogens in plants: life up against the wall Plant Cell 1996 8 1683 1698 10.1105/tpc.8.10.1683 12239358 PMC161307

[B6] Rahme LG Mindrinos MN Panopoulos NJ Plant and environmental sensory signals control the expression of *hrp *genes in *Pseudomonas syringae *pv. phaseolicola J Bacteriol 1992 174 3499 3507 1592805 10.1128/jb.174.11.3499-3507.1992 PMC206034

[B7] Aldon D Brito B Boucher C Genin S A bacterial sensor of plant cell contact controls the transcriptional induction of *Ralstonia solanacearum *pathogenicity genes EMBO Journal 2000 19 2304 2314 10.1093/emboj/19.10.2304 10811621 PMC384368

[B8] Mo YY Gross DC Plant signal molecules activate the *syrB *gene, which is required for syringomicin production by *Pseudomonas syringae *pv. syringae J Bacteriol 1991 173 5784 5792 1885550 10.1128/jb.173.18.5784-5792.1991 PMC208311

[B9] Li XZ Starratt AN Cuppels DA Identification of tomato leaf factors that activate toxin gene expression in *Pseudomonas syringae *pv. tomato DC3000 Phytopathol 1998 88 1094 1100 10.1094/PHYTO.1998.88.10.1094 18944822

[B10] Kelemu S Collmer A *Erwinia chrysantemi *EC16 produces a second set of plant-inducible pectate lyase isoenzymes Appl Environ Microbiol 1993 59 1756 1761 16348952 10.1128/aem.59.6.1756-1761.1993 PMC182157

[B11] Lindgren PZ Peet RC Panopoulus NJ Gene cluster of *Pseudomonas syringae *pv "phaseolicola" controls pathogenicity of bean plants and hypersensitivity on nonhost plants J Bacteriol 1986 168 512 522 3023280 10.1128/jb.168.2.512-522.1986 PMC213511

[B12] Schwartz HF Bacterial diseases of beans Crop series diseases no 2.913 2001 http://www.ext.colostate.edu/crops/02913.pdf

[B13] Brencic A Winans SC Detection of and response to signals involved in host-microbe interactions by plant-associated bacteria Microbiol Mol Biol Rev 2005 69 155 194 10.1128/MMBR.69.1.155-194.2005 15755957 PMC1082791

[B14] Rico A Preston GM *Pseudomonas syringae *pv. tomato DC3000 uses constitutive and apoplast-induced nutrient assimilation pathways to catabolize nutrients that are abundant in the tomato apoplast Mol Plant-Microbe Interact 2008 21 269 282 10.1094/MPMI-21-2-0269 18184070

[B15] Lan L Deng X Zhou J Tang X Genome-wide gene expression analysis of *Pseudomonas syringae *pv. tomato DC3000 reveals overlapping and distinct pathways regulated by *hrpL *and *hrpRS* Mol Plant-Microbe Interact 2006 19 976 987 10.1094/MPMI-19-0976 16941902

[B16] Gibson G Wolfinger R Myron SA, Balzarini MG, Cappio-Borlino A Gene expression profiling using mixed models Genetics Analysis of Complex Traits Using SAS 2004 Cary, NC, USA: SAS Press 251 278

[B17] Wolfinger RD Gibson G Wolfinger ED Bennett L Hamadeh H Bushel P Afshari C Paules RS Assessing gene significance from cDNA microarray expression data via mixed models J Comput Biol 2001 8 6 625 637 10.1089/106652701753307520 11747616

[B18] Eisen MB Spellman PT Brown PO Botstein D Cluster analysis and display of genome-wide expression patterns Proc Natl Acad Sci 1998 95 25 14863 14868 10.1073/pnas.95.25.14863 9843981 PMC24541

[B19] Schjoerring JK Pearson N Husted S Nielsen KH Mattsson M Horst WJ The leaf apoplast: a central compartment in plant nitrogen utilization Plant Nutrition-Food security and sustainability of agro-ecosystems through basic and applied research. Netherlands 2001 92 224 245

[B20] Solomon PS Oliver RP The nitrogen content of the tomato leaf apoplast increases during infection by *Cladosporium fulvum* Planta 2001 213 241 249 10.1007/s004250000500 11469589

[B21] Joosten MHAJ Hendrickx LJM De Wit PJGM Carbohydrate composition of apoplastic fluids isolated from tomato leaves inoculated with virulent or avirulent races of *Cladosporium fulvum *(syn. *Fulvia fulva*) Neth J Pl Path 1990 96 103 112 10.1007/BF02005134

[B22] Mattinen L Somervuo P Nykyri J Nissinen R Kuovonen P Corthals G Auvinen P Aittamaa M Valkonen JP Pirhonen M Microarray profiling of host-extract-induced genes and characterization of the type VI secretion cluster in the potato pathogen *Pectobacterium atrosepticum* Microbiology 2008 154 2387 2396 10.1099/mic.0.2008/017582-0 18667571

[B23] Joardar V Lindeberg M Jackson RW Selengut J Dodson R Brinkac LM Daugherty SC DeBoy R Durkin AS Giglio MG Madupu R Nelson WC Rasovitz MJ Sullivan S Crabtree J Creasy T Davidsen T Haft DH Zafar N Zhou L Halpin R Holley T Khouri H Feldblyum T White O Fraser CM Chatterjee AK Cartinhour S Schneider DJ Mansfield J Collmer A Buell R Whole genome sequence analysis of *Pseudomonas syringae *pv phaseolicola 1448A reveals divergence among pathovars in genes involved in virulence and transposition J Bacteriol 2005 187 6488 6498 10.1128/JB.187.18.6488-6498.2005 16159782 PMC1236638

[B24] Hueck CJ Type III protein secretion systems in bacterial pathogens of animals and plants Microbiol Mol Biol Rev 1998 62 379 433 9618447 10.1128/mmbr.62.2.379-433.1998 PMC98920

[B25] Collmer A Badel JL Charkowski AO Deng WL Fouts DE Ramos AR Rehm AH Anderson DM Schneewind O van Dijk K Alfano JR *Pseudomonas syringae *Hrp type III secretion system and effector proteins Proc Natl Acad Sci 2000 97 8770 8777 10.1073/pnas.97.16.8770 10922033 PMC34010

[B26] Kunkel BN Chen Z Virulence strategies of plant pathogenic bacteria Prokaryotes 2006 2 421 440 full_text

[B27] Okinaka YYC Perna NT Keen NT Microarray profiling of *Erwinia chrysanthemi *3937 genes that are regulated during plant infection Mol Plant-Microbe Interact 2002 15 7 619 629 10.1094/MPMI.2002.15.7.619 12118877

[B28] Mattinen L Nissinen R Riipi T Kalkkinen N Pirhonen M Host-extract induced changes in the secretome of the plant pathogenic bacterium *Pectobacterium atrosepticum* Proteomics 2007 7 3527 3537 10.1002/pmic.200600759 17726675

[B29] Collmer A Keen NT The role of pectic enzymes in plant pathogenesis Annu Rev Phytopathol 1986 24 383 409 10.1146/annurev.py.24.090186.002123

[B30] Perombelon MCM Potato diseases caused by soft rot Erwinias: an overview of pathogenesis Plant Pathol 2002 51 1 12 10.1046/j.0032-0862.2001.Short title.doc.x

[B31] Salmond GPC Secretion of extracellular virulence factors by plant-pathogenic bacteria Annu Rev Phytopathol 1994 32 181 200 10.1146/annurev.py.32.090194.001145

[B32] Longland AC Slusarenko AJ Friend J Pectolytic enzymes from interactions between *Pseudomonas syringae *pv. phaseolicola and French bean (*Phaseolus vulgaris*) J Phytopathol 1992 134 75 86 10.1111/j.1439-0434.1992.tb01214.x

[B33] Magro P Varvaro L Chilosi G Avanzo C Balestra GM Pectolytic enzymes produced by *Pseudomonas syringae *pv. glycinea FEMS Microbiol Lett 1994 117 1 6 10.1111/j.1574-6968.1994.tb06733.x

[B34] Charkowski AO Alfano JR Preston G Yuan J He SY Collmer A The *Pseudomonas syringae *pv. tomato HrpW protein has domains similar to harpins and pectate lyases and can elicit the plant hypersensitive response and bind to pectate J Bacteriol 1998 180 5211 5217 9748456 10.1128/jb.180.19.5211-5217.1998 PMC107559

[B35] Kvitko BH Ramos AR Morello JE Oh HS Collmer A Identification of harpins in *Pseudomonas syringae *pv. tomato DC3000, which are functionally similar to HrpK1 in promoting translocation of type III secretion system effectors J Bacteriol 2007 189 8059 8072 10.1128/JB.01146-07 17873033 PMC2168707

[B36] Vencato M Tian F Alfano JR Buell CR Cartinhour S DeClerck GA Guttman DS Stavrinides J Joardar V Lindeberg M Bronstein PA Mansfield JW Myers CR Collmer A Schneider DJ Bioinformatics-enabled identification of the HrpL regulon and type III secretion system effector proteins of *Pseudomonas syringae *pv. phaseolicola 1448A Mol Plant-Microbe Interact 2006 19 1193 1206 10.1094/MPMI-19-1193 17073302

[B37] Idriss EE Makarewicz O Farouk A Rosner K Greiner R Bochow H Richter T Borriss R Extracellular phytase activity of *Bacillus amyloliquefaciens *FZB45 contributes to its plant-growth-promoting effect Microbiol 2002 148 2097 2109 10.1099/00221287-148-7-2097 12101298

[B38] Vohra A Satyanarayana T Phytases: microbial sources, production, purification, and potential biotechnological applications Critical Reviews in Biotechnology 2003 23 29 60 10.1080/713609297 12693443

[B39] Dave OB Blanchard C Balasubramanian P Phytic acid, phytase, minerals, and antioxidant activity in Canadian dry bean (*Phaseolus vulgaris *L.) cultivars J Agric Food Chem 2008 56 11312 11319 10.1021/jf801661j 18989970

[B40] Rathmell WG Sequeira L Soluble peroxidase in fluid from the intercellular spaces of tobacco leaves Plant Phyisol 1974 53 317 318 10.1104/pp.53.2.317 PMC541386 16658698

[B41] Aguilera S López-López K Nieto Y Garcidueñas-Piña R Hernández-Guzmán G Hernández-Flores JL Murillo J Álvarez-Morales A Functional characterization of the gene cluster from *Pseudomonas syringae *pv. phaseolicola NPS3121 involved in synthesis of phaseolotoxin J Bacteriol 2007 189 2834 2843 10.1128/JB.01845-06 17237165 PMC1855804

[B42] Quigley NB Gross DC Syringomicin production among strains of *Pseudomonas syringae *pv. syringae: conservation of the *syrB *and *syrD *genes and activation of phytotoxin production by plant signal molecules Mol Plant-Microbe Interact 1994 7 78 90 7909458 10.1094/mpmi-7-0078

[B43] Mo YY Geibel M Bonsall RF Gross DC Analysis of sweet cherry (*Prunus avium *L.) leaves for plant signal molecules that activate the syrB gene requires for synthesis of the phytotoxin, syringomycin, by *Pseudomonas syringae *pv. syringae Plant Physiol 1995 107 603 612 12228385 10.1104/pp.107.2.603 PMC157164

[B44] Mosqueda G Den Broeck GV Saucedo O Bailey AM Alvarez-Morales A Herrera-Estrella L Isolation and characterization of the gene from *Pseudomonas syringae *pv. phaseolicola encoding the phaseolotoxin-insensitive ornithine carbamoyltransferase Mol Genet 1990 222 461 466 10.1007/BF00633857 2274044

[B45] López-López K Hernández-Flores JL Cruz-Aguilar M Alvarez-Morales A In *Pseudomonas syringae *pv. phaseolicola the phaseolotoxin-resistant ornithine carbamoyltransferase encoded by *argK *is indirectly regulated by temperature and directly by a precursor molecule resembling carbamoylphospate J Bacteriol 2004 186 146 153 10.1128/JB.186.1.146-153.2004 14679234 PMC303443

[B46] Rico A Jones R Preston GM Jackson RW Adaptation to the plant apoplast by plant pathogenic bacteria Plant Pathogenic Bacteria: Genomics and Molecular Biology 2009 School of Biological Sciences, University of Reading, Whiteknights, Reading, UK 63 89

[B47] Herrera-Flores TS Cárdenas-Soriano E Ortíz-Cereceres J Acosta-Gallegos JA Mendoza-Castillo MC Anatomy of the pod of three species of the genus Phaseolus Agrociencia 2005 39 595 602

[B48] Brandt U Energy converting NADH:Quinone Oxidoreductase (Complex 1) Annu Rev Biochem 2006 75 69 92 10.1146/annurev.biochem.75.103004.142539 16756485

[B49] Okuda S Katayama T Kawashima S Goto S Kanehisa M ODB: a database of operons accumulating known operons across multiple genomes Nucleic Acids Res 2006 D358 D362 10.1093/nar/gkj037 16381886 PMC1347400

[B50] Lund PA Microbial molecular chaperones Adv Microb Phyisiol 2001 44 93 140 full_text 10.1016/s0065-2911(01)44012-4 11407116

[B51] Zwiesler-Vollick J Plovanich-Jones A Nomura K Bandyopadhyay S Joardar V Kunkel BN He SY Identification of novel hrp-regulated genes through functional genomic analysis of the Pseudomonas syringae pv tomato DC3000 genome Mol Microbiol 2002 45 1207 1218 10.1046/j.1365-2958.2002.02964.x 12207690

[B52] Klotz MG Hutcheson SW Multiple periplasmic catalases in phytopathogenic strains of *Pseudomonas syringae* Appl Environ Microbiol 1992 58 2468 2473 1514792 10.1128/aem.58.8.2468-2473.1992 PMC195805

[B53] Andrews SC Robinson AK Rodríguez-Quiñones F Bacterial iron homeostasis FEMS Microbiol Rev 2003 27 215 237 10.1016/S0168-6445(03)00055-X 12829269

[B54] Ma JF Ochsner UR Klotz MG Nanayakkara VK Howell ML Johnson Z Posey JE Vasil ML Monaco JJ Hassett DJ Bacterioferritin A modulates catalase A (*KatA*) activity and resistance to hydrogen peroxide in *Pseudomonas aeruginosa* J Bacteriol 1999 181 3730 3742 10368148 10.1128/jb.181.12.3730-3742.1999 PMC93851

[B55] Vasil ML How we learnt about iron acquisition in *Pseudomonas aeruginosa *: a series of very fortunate events Biometals 2007 20 587 601 10.1007/s10534-006-9067-2 17186376

[B56] Llamas MA Mooij MJ Sparrius M Vandenbroucke-Grauls CM Ratledge C Bitter W Characterization of five novel *Pseudomonas aeruginosa *cell-surface signalling systems Mol Microbiol 2008 62 7 458 472 10.1111/j.1365-2958.2007.06061.x 18086184

[B57] Swingle B Thete D Moll M Myers CR Schneider DJ Cartinhour S Characterization of the PvdS-regulated promoter motif in *Pseudomonas syringae *pv. tomato DC3000 reveals regulon members and insights regarding PvdS function in other pseudomonads Mol Microbiol 2008 68 4 871 889 10.1111/j.1365-2958.2008.06209.x 18363796

[B58] Feil H Feil WS Chain P Larimer F DiBartolo G Copeland A Lykidis A Trong S Nolan M Goltsman E Thiel J Malfatti S Loper JE Lapidus A Detter JC Land M Richardson PM Kyrpides NC Ivanova N Lindow SE Comparison of the complete genome sequences of *Pseudomonas syringae *pv. syringae B728a and pv. tomato DC3000 Proc Natl Acad Sci 2005 102 11064 11069 10.1073/pnas.0504930102 16043691 PMC1182459

[B59] Jones AM Lindow SE Wildermuth MC Salicylic acid, yersiniabactin, and pyoverdine production by the model phytopathogen *Pseudomonas syringae *pv. tomato DC Synthesis, regulation, and impact on tomato and *Arabidopsis *host plants J Bacteriol 3000 189 19 6773 6786 10.1128/JB.00827-07 PMC2045226 17660289

[B60] Braun V Braun M Iron transport and signaling in *Escherichia coli* FEBS Letters 2002 529 78 85 10.1016/S0014-5793(02)03185-X 12354617

[B61] Leoni L Orsi N de Lorenzo V Visca P Functional analysis of PvdS, an iron starvation sigma factor of *Pseudomonas aeruginosa* J Bacteriol 2000 182 6 1481 1491 10.1128/JB.182.6.1481-1491.2000 10692351 PMC94443

[B62] Wilderman PJ Sowa NA FitzGerald DJ FitzGerald PC Gottesman S Ochsner UA Vasil ML Identification of tandem duplicate regulatory small RNAs in *Pseudomonas aeruginosa *involved in iron homeostasis Proc Natl Acad Sci 2004 101 26 9792 9797 10.1073/pnas.0403423101 15210934 PMC470753

[B63] Chen WP Kuo TT A simple and rapid method for the preparation of gram negative bacterial genomic DNA Nucleic Acids Res 1993 21 2260 10.1093/nar/21.9.2260 8502576 PMC309503

[B64] De Ita ME Marsch-Moreno R Guzmán P Álvarez-Morales A Physical map of chromosome of the phytophatogenic bacterium *Pseudomonas syringae *pv. phaseolicola Microbiology 1998 144 493 501 10.1099/00221287-144-2-493 33757227

[B65] The R project for statistical computing http://www.r-project.org

[B66] Irizarry RA Bolstad BM Collin F Cope LM Hobbs B Speed TP Summaries of Affymetrix, GeneChip probe level data Nucleic Acid Res 2003 31 4 e15 10.1093/nar/gng015 12582260 PMC150247

[B67] Yang YH Dudoit S Luu P Lin DM Peng V Ngai J Speed TP Normalization for cDNA microarray data: a robust composite method addressing single and multiple slide systematic variation Nucleic Acid Res 2002 30 4 e15 10.1093/nar/30.4.e15 11842121 PMC100354

[B68] Limma: linear models for microarray data user's guide http://www.bioconductor.org

[B69] Benjamini Y Hochberg Y Controlling the False Discovery Rate: A practical and powerful approach to multiple testing J R Statist Soc B 1995 57 289 300

